# Initial Psychometric Evaluation of the Barth Syndrome Symptom Assessment (BTHS-SA) for Adolescents and Adults in a Phase 2 Clinical Study

**DOI:** 10.1186/s13023-025-03693-5

**Published:** 2025-04-25

**Authors:** Chad Gwaltney, Alan Shields, Emily Love, Sarah Ollis, Jonathan Stokes, Iyar Mazar, Ethan Arenson, Anthony Aiudi, R. J. Wirth, Carrie Houts

**Affiliations:** 1 Gwaltney Consulting Group, Westerly, RI USA; 2 Present Address: Adelphi Values, Boston, MA USA; 3 Stealth BioTherapeutics, Needham, MA USA; 4 Vector Psychometric Group, Chapel Hill, NC USA

**Keywords:** Barth syndrome, Barth syndrome symptom assessment, BTHS-SA, Instrument development, Patient-reported outcome, PRO, Psychometric evaluation

## Abstract

**Background:**

Barth syndrome (BTHS) is a rare, X-linked disorder that stems from mutations in the *TAFAZZIN *(TAZ) gene with varying disease severity among patients. The Barth Syndrome Symptom Assessment (BTHS-SA) is a patient-reported outcome questionnaire developed to assess BTHS symptom severity. The current study reflects the first exploration of the assessment’s psychometric performance.

**Methods:**

The BTHS-SA was administered in TAZPOWER, a phase 2, randomized, double-blind, placebo-controlled crossover study to evaluate daily subcutaneous injections of elamipretide in subjects with genetically confirmed BTHS. Descriptive and correlational analyses were used to assess the score distributions, reliability, and construct-related validity of BTHS-SA items and domains including a two-item (2 FS), three-item (3 FS), and four-item (4 FS) fatigue score, and a five-item myopathy score (5MS).

**Results:**

Among the N = 12 white males (M age = 19.5, SD = 7.7) participating in the TAZPOWER trial, overall symptoms were rated as mild (n = 5, 41.7%), moderate (n = 5, 41.7%), severe (n = 1, 8.3%), or very severe (n = 1, 8.3%). Descriptive statistics for the BTHS-SA scores indicate variability of symptom severity both within symptom cluster and across patients. Promising results were found for both internal consistency (α = 0.67, 0.72, and 0.66 for the 3 FS, 4 FS, and 5MS, respectively) and test–retest reliability (ICC values ranging from 0.79 to 0.94 across two test–retest intervals). Correlational analyses showing moderate to strong relationships to other patient reports of fatigue (e.g., r = 0.59, 0.76, 0.68, and 0.61 between the PROMIS Fatigue SF and the 2 FS, 3 FS, 4 FS, and 5MS, respectively) and symptom severity (e.g., r = 0.60, 0.62, 0.56, 0.53 between a patient global rating and the 2 FS, 3 FS, 4 FS, and 5MS, respectively) support the measure’s convergent validity. A similar pattern of relationships was observed when correlating changes in BTHS-SA scores to reference measures, including moderate to strong relationships between the BTHS-SA and direct patient reports of change (r = 0.81, 0.79, 0.82, and 0.80 between a global impression of change score and the 2 FS, 3 FS, 4 FS, and 5MS, respectively).

**Conclusion:**

Though the small sample size limits strong conclusions, this analysis suggests the BTHS-SA can produce reliable scores upon which valid inferences may be drawn. The BTHS-SA may be a useful tool to evaluate treatment benefits in this underserved population.

***Trial registration*:**

ClinicalTrials.gov identifier, NCT03098797. Registered 05 May 2017, https://www.clinicaltrials.gov/study/NCT03098797.

## Background

Barth syndrome (BTHS) is a rare, life-threatening, X-linked disorder that stems from mutations in the *TAFAZZIN* gene (*TAZ*, *G4.5*) resulting in abnormal cardiolipin on the inner mitochondrial membrane, with a prevalence of approximately 1 in 1,000,000 male births [1–4]. Clinical signs and symptoms of BTHS often present in infancy and manifest clinically as cardiomyopathy (leading to arrhythmia and/or congestive heart failure), neutropenia (leading to infections), growth and motor function delay, and skeletal muscle myopathy (leading to weakness, exercise intolerance, and fatigue) [5–8]. Disease severity can be highly variable between individuals, with the range of symptoms associated with BTHS having the potential to negatively impact individuals'overall quality-of-life including social, physical, emotional, and daily functioning [5, 7, 9]. Currently, there are no approved disease-specific treatments available for BTHS, so specific symptoms are monitored and treated as they are experienced [2, 10–12]. Disease-specific treatments are being studied in clinical trials [2].

There is a lack of well-defined and reliable measures of disease symptomology to assess the potential clinical benefits of novel treatments for BTHS [13]. In the development of new treatments, it is important to consider outcomes that are relevant to the disease and important to patients; reduction of symptoms is a potentially critical outcome that could improve patient quality of life and reduce burden on the clinical care system. Accordingly, a novel, BTHS-specific symptom questionnaire was created—the Barth Syndrome Symptom Assessment (BTHS-SA) [13]. The content of the BTHS-SA was based on patient and caregiver input and created in alignment with best practices for patient-reported outcome (PRO) development [14–16].

With its content validity established through qualitative patient interviews, the BTHS-SA was subsequently administered to patients enrolled in TAZPOWER, the first clinical trial to evaluate the safety and efficacy of a novel, disease-specific treatment for patients with BTHS [17–19]. The goal of the current analyses was to descriptively explore the reliability and validity of the BTHS-SA scores collected in the double-blind portion (Part 1) of the TAZPOWER study.

## Methods

### Study design

**Fig. 1:**
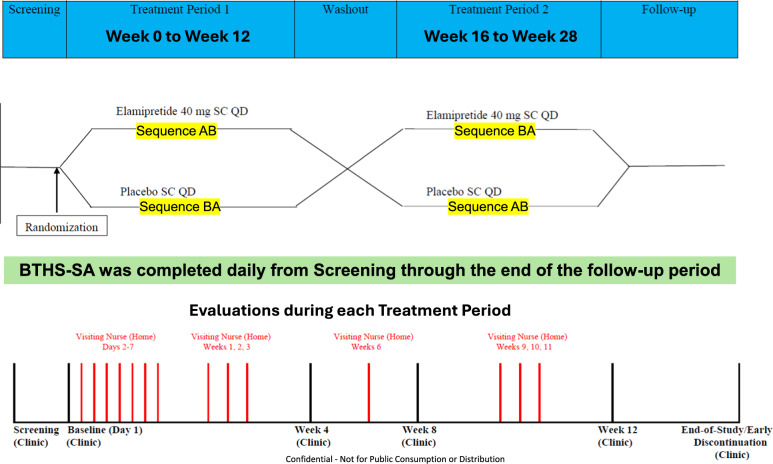
TAZPOWER study design schematic

The TAZPOWER study (Fig. 1) was a 28-week Phase 2, randomized, double-blind, placebo-controlled crossover study to evaluate daily subcutaneous injections of elamipretide in subjects with genetically-confirmed BTHS [17–19]. The double-blind phase (Part 1) consisted of two 12-week treatment periods (Period 1 and Period 2) separated by a 4-week washout period. Patients were randomized to Sequence AB (elamipretide in Period 1 and placebo in Period 2) or Sequence BA (placebo in Period 1 and elamipretide in Period 2).

### Analysis population

All subjects met all of the inclusion criteria, which broadly included North American male consenting adolescents and adults (aged ≥ 12 years) with genetically-confirmed BTHS who were ambulatory and impaired during the Six-Minute Walk Test (6MWT) [17]. Subjects with medical conditions that could put them at risk, who had been hospitalized within 30 days prior to baseline, had uncontrolled hypertension, or who were actively enrolled in another trial within 30 days prior to baseline were excluded [17].

### Study assessments

#### BTHS-SA

**Fig. 2:**
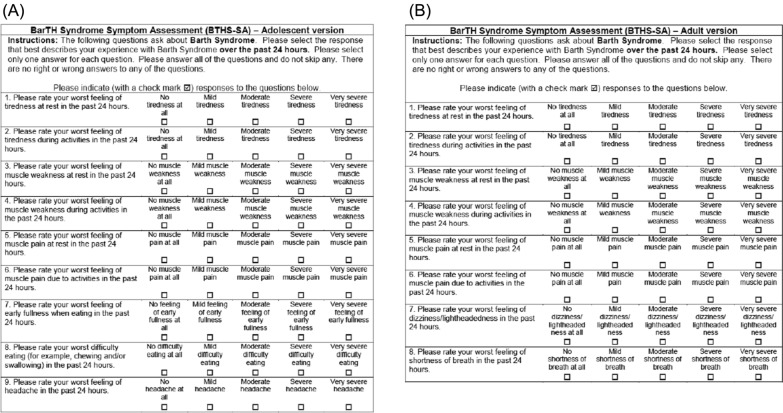
BTHS-SA **a** adolescent (16 years) and **B** adult (≥ 16 years) versions

Subjects completed the electronic BTHS-SA diary on each day of the study (between 6:00 pm and 11:59 pm) beginning at Screening and through the end of the follow-up period or until early discontinuation [17]. The BTHS-SA was created with two versions, one for use among adolescents ages 12–15 years (9 items) and the other for adults ≥ 16 years old (8 items) with BTHS (Fig. 2) [13, 17]. Both versions of the BTHS-SA use a 24-h recall period and ask patients to rate the severity of tiredness, muscle weakness, and muscle pain (each independently at rest and during activities) on a five-point verbal response scale (ranging from 1 [Not at all] to 5 [Very severe]) [13]

.

As an assessment in early development, there may be a variety of ways in which the BTHS-SA may be scored. In the present analysis, assessment domains were created using the shared items 1 through 5 (i.e., those items administered exactly the same to adolescents and adults). Specifically, three fatigue domains were hypothesized including a two-item fatigue score (2 FS, the sum of the tiredness and muscle weakness during activities items); three-item fatigue score (3 FS, the sum of the tiredness and muscle weakness during activities items and the tiredness at rest item); and four-item fatigue score (4 FS, the sum of the tiredness and muscle weakness during activities and at rest items). Additionally, a five-item myopathy score (5MS) was derived as the sum of items used for the 4 FS and the muscle pain at rest item.

For both the efficacy analysis, as well as the psychometric analysis described here, weekly scores were derived as the average of daily values collected on the seven days preceding a target analysis day. For example, if the Baseline visit (Day 1) is the target analysis day then the Baseline weekly score is the average of scores generated from study Days 0, − 1, − 2, − 3, − 4, − 5, and − 6. A daily score required at least 70% of the items be non-missing (e.g., 4 out of 5 items for the 5MS, 3 out of 4 items for the 4 FS, and all items for each of the 2 FS and 3 FS). For days with enough answered items, a pro-rated summed score was calculated as:

$${\text{Pro - rated}}\,{\text{summed}}\,{\text{score}}\,{ = }\,\frac{{{\text{(summed}}\,{\text{score}}\,{\text{of}}\,{\text{answered}}\,{\text{items}}\,*\,{\text{total}}\,{\text{number}}\,{\text{of}}\,{\text{items}})}}{{\left( {{\text{numbered}}\,{\text{of}}\,{\text{answered}}\,{\text{items}}} \right)}}$$

Subsequently, if four or more of the daily BTHS-SA scores were missing in the specified weekly interval, the weekly score was treated as missing.

#### Supportive measures

Subjects were asked to complete additional assessments at study visits during a seven-day Screening period, at Baseline of each treatment period (Week 1 [Visit 1] for treatment period 1 and Week 17 [Visit 6] for treatment period 2), Week 12 (Visit 5), Week 17 (Visit 6), and Week 28 (Visit 10) [17]. These additional assessments included the Patient Reported Outcome Measurement Information System (PROMIS) Fatigue short form [20]; the EQ- 5D- 5L [21]; Patient Global Impression of Severity (PGI-S) and Impression of Change (PGI-C), Clinician Global Impression of Severity (CGI-S) and Impression of Change (CGI-C) [22, 23], and Caregiver Global Impression of Severity (CaGI-S) and Impression of Change (CaGI-C) [17]. Global Impression of Severity items asked the respondent to rate the severity of the patient’s BTHS-SA symptoms in the past week. Global Impression of Change items asked the respondent to rate the change in the patient’s symptoms since the start of each treatment period. Each of the global items asked respondents to consider their overall symptoms collectively (“your Barth Syndrome symptoms”).

Patients also completed functional assessments including the 6MWT [24], which measures the distance, in meters, that a subject covers during a six-minute period and two self-report items assessing shortness of breath and fatigue before and after the 6MWT (12-point modified Borg scale); the Five Times Sit-to-Stand Test (5XSST) [25], which measures the time in seconds to stand up 5 times, without stopping in between sittings [17]; and the SWAY Application Balance Assessment (SWAY) [26] that provides a numerical quantification of postural sway, with 0 being unstable and 100 being completely stable [17]. Handheld dynamometer (HHD) data [27] was used to assess leg strength via the “make technique”, in which the tester matched the subject’s maximum isometric contraction for five seconds [17]. These measures are described in detail in the TAZPOWER publications [17–19].

#### Analyses

All analyses were conducted in SAS 9.4 and focused on an initial descriptive evaluation of the performance of BTHS-SA 2 F, 3 F, 4 F, and 5MS domains which consisted of the items shared by both the adult and adolescent versions of the assessment.

#### Descriptive statistics

The BTHS-SA was administered daily in the study and here descriptive statistics are presented for the weekly 2 FS, 3 FS, 4 FS, and 5MS as well as the items that contribute to those domains, collected at the Pre-dose Visit (i.e., Days − 6 to 0 or the 7 days prior to the Baseline visit), Nurse Visit 2 (i.e., the 7 days prior to the Nurse Visit 2), and End of Treatment Period Visit (i.e., the 7 days prior to the End of Treatment Period visit).

#### Reliability analysis

Reliability estimates characterize consistency and reproducibility of a particular set of scores, and in this study, was assessed in two ways. First, Cronbach’s coefficient alpha (α) was computed to generate internal consistency estimates of the 2 FS, 3 FS, 4 FS, and 5MS at each of the daily analysis time points (i.e., Day − 6, Day 1, and every subsequent 18 th day [Days 19, 37, and up to Day 199]) and descriptively analyzed via mean and median across the time intervals. Second, test–retest correlations, both Pearson correlation and intraclass correlation coefficients (ICCs) from a two-way mixed effects model with absolute agreement for single measures [28], were calculated for two time periods based on the expectation that subjects have relatively stable health status within these time frames:

- Time Period 1: between Screening week 1 (Day − 13 to Day − 7) and Screening week 2 (Day − 6 to Day 0).
- Time Period 2: between the last two weeks of the washout period, prior to the start of treatment period 2 (Week 16 and Week 17 on the study schedule).

#### Construct-related validity analysis

Construct-related validity conclusions can be based on the magnitude of observed relationship between scores produced by a target questionnaire and reference measures. In other words, logical relationships ought to exist between measures that reflect characteristics of patients (e.g., measures of pain ought to be more strongly related to each other than to, say, measures of more distal concepts such as well-being). Using correlational methods, construct-related validity of the BTHS-SA was evaluated in two ways. First, correlation coefficients were generated for available variables at Visits 1 through 5 for Treatment Period 1 and at Visits 6 through 10 for Treatment Period 2 and then averaged. Second, as indicators of sensitivity-to-change (a type of construct-related validity), correlation coefficients were generated between change in weekly BTHS-SA scores with change scores observed in the relevant reference measures. In both instances, Pearson’s correlations were generated for continuous variables and Spearman’s correlation for categorical variables.

## Results

### Sample

Twelve males participated in both treatment periods of the double-blind phase of the TAZPOWER clinical trial. Participants had a mean age of 19.5 (SD = 7.7) years at screening for Treatment Sequence AB (elamipretide-placebo) and a mean age of 20.3 (SD = 7.3) at screening for Treatment Sequence BA (placebo-elamipretide). Half of the sample were between 12 and 16 years of age, with the other half of the sample being between 17 and 35 years of age. All participants self-identified as non-Hispanic white, though one participant dually self-identified as American Indian or Alaskan Native. At the screening visit patients (n = 12) self-reported their overall Barth syndrome related symptoms as mild (n = 5, 41.7%), moderate (n = 5, 41.7%), severe (n = 1, 8.3%), or very severe (n = 1, 8.3%).

### BTHS-SA score descriptives

The BTHS-SA 2 FS, 3 FS, 4 FS, and 5MS and the items included in each are presented in Table 1 for the Pre-Dose, Nurse Visit 2 (Week 1, study Day 8 ± 1), and End of Treatment Period 1 visits. On average, subjects reported feelings of tiredness, both at rest (Item 1) and during activities, more severely than their other symptoms across timepoints. As expected, subjects also consistently reported experiencing more severe symptoms during activities than at rest.

**Table 1:** BTHS-SA Weekly item and domain scores

Scale/item	Treatment period 1					
	Pre-dose		Nurse visit 2		End of Tx	
	N	M (SD)	N	M (SD)	N	M (SD)
2 FS (range = 2–10)	12	5.42 (0.98)	12	4.11 (1.21)	12	4.22 (1.04)
3 FS (range = 3–15)	12	8.0 (1.35)	12	6.09 (1.95)	12	6.26 (1.69)
4 FS (range = 4–20)	12	9.96 (1.65)	12	7.60 (2.44)	12	7.96 (2.14)
5MS (range = 5–25)	12	11.84 (1.76)	12	9.27 (2.61)	12	9.85 (2.64)
Item 1: tiredness at rest (range = 1–5)	12	2.58 (0.84)	12	1.98 (0.85)	12	2.04 (0.83)
Item 2: tiredness during activity (range = 1–5)	12	2.85 (0.64)	12	2.17 (0.81)	12	2.25 (0.58)
Item 3: muscle weakness at rest (range = 1–5)	12	1.96 (0.48)	12	1.52 (0.51)	12	1.70 (0.62)
Item 4: muscle weakness during activity (range = 1–5)	12	2.57 (0.49)	12	1.94 (0.51)	12	1.97 (0.59)
Item 5: muscle pain at rest (range = 1–5)	12	1.67 (0.52)	12	1.47 (0.59)	12	1.69 (0.63)

Scale/item	Treatment period 2					
	Pre-dose		Nurse visit 2		End of Tx	
	N	M (SD)	N	M (SD)	N	M (SD)
2 FS (range = 2–10)	12	4.73 (1.09)	11	4.39 (1.24)	11	4.20 (1.29)
3 FS (range = 3–15)	12	7.10 (1.85)	11	6.65 (1.85)	11	6.28 (2.02)
4 FS (range = 4–20)	12	8.94 (2.21)	11	8.26 (2.24)	11	7.78 (2.35)
5MS (range = 5–25)	12	11.20 (2.66)	11	10.13 (2.71)	11	9.54 (3.03)
Item 1: tiredness at rest (range = 1–5)	12	2.38 (0.96)	11	2.26 (0.94)	11	2.08 (0.99)
Item 2: tiredness during activity (range = 1–5)	12	2.57 (0.83)	11	2.47 (0.99)	11	2.23 (0.90)
Item 3: muscle weakness at rest (range = 1–5)	12	1.83 (0.64)	11	1.61 (0.69)	11	1.50 (0.48)
Item 4: muscle weakness during activity (range = 1–5)	12	2.15 (0.52)	11	1.92 (0.58)	11	1.97 (0.70)
Item 5: muscle pain at rest (range = 1 to 5)	12	1.94 (0.74)	11	1.61 (0.67)	11	1.50 (0.58)

2 FS = 2 item fatigue during activities score; 3 FS = 3 item fatigue score; 4 FS = 4 item fatigue score; 5MS = 5 item myopathy score

### Reliability analyses

#### Internal consistency

Averaged across timepoints, median and mean values for Cronbach’s α for the 2 FS (0.59, 0.53), 3 FS (0.67, 0.62), 4 FS (0.72, 0.65), and 5MS (0.66, 0.66) suggest promising levels of item interrelatedness for the scores with 3 or more items that support their use in clinical research (i.e., while there are no universally accepted rules for the interpretation of α, estimates of approximately 0.70 have been regarded as sufficient when the measurement scores are used for group-level research purposes and particularly early in questionnaire development) [28].

#### Test–retest reliability

Results displayed in Table 2 shows evidence of BTHS-SA scale score reproducibility over time, with ICC estimates ranging from 0.79 to 0.94 across the two test–retest intervals. Despite small sample sizes, it is reasonable to conclude that scale scores were similar over two test–retest periods during which significant change in health status was not anticipated.

**Table 2:** Test–retest reliabilities of BTHS-SA scale scores

Scale	Time period 1*			Time period 2*		
	N	r	ICC	N	r	ICC
2 FS	11	0.87	0.84	12	0.93	0.93
3 FS	11	0.86	0.85	12	0.95	0.94
4 FS	11	0.81	0.79	12	0.94	0.94
5MS	11	0.88	0.81	12	0.89	0.88

*Pearson correlation (r) and intraclass correlation coefficients (ICCs) from a two-way mixed effects model with absolute agreement for single measures [28] were calculated for each time period

#### Construct-related validity analyses

The correlational results in Table 3 are presented to evaluate the observed relationships between the BTHS-SA scale scores and the reference measures using the following guidelines:/r/= 0.0 to 0.30, 0.31 to 0.70, 0.71 to 0.90, and 0.91 to 1.00 = weak, moderate, strong, and very strong relationship, respectively [29]. Though correlations can be very unstable with small sample sizes, an overall pattern of both convergent and discriminant validity emerged.

**Table 3:** Correlations for BTHS-SA scores and reference measures averaged across timepoints

	BTHS-SA scale			
	2 FS	3 FS	4 FS	5MS
PROMIS Fatigue SF*	0.59	0.76	0.68	0.61
6MWT*	− 0.30	− 0.47	− 0.52	− 0.51
EQ5D VAS*	− 0.28	− 0.34	− 0.32	− 0.23
5XSST (secs to complete)*	0.27	0.28	0.06	0.08
SWAY*	− 0.12	− 0.05	− 0.28	0.02
Activity duration*	− 0.29	− 0.26	− 0.13	− 0.09
Activity intensity*	− 0.31	− 0.29	− 0.14	− 0.10
CGI-S**	0.40	0.40	0.54	0.36
PGI-S**	0.60	0.62	0.56	0.53
CaGI-S**	0.46	0.47	0.41	0.43
6MWT PRO fatigue (post)**	0.64	0.57	0.58	0.56
6MWT PRO Dyspnea (post)**	0.63	0.67	0.69	0.64

To facilitate interpretation, each of the 2 FS (range = 2–10), 3 FS (range = 3–15), 4 FS (range = 4–20), and 5MS (range = 5–25) are scored such that higher scores reflect poorer health status; PROMIS Fatigue SF (range = 0–52) is scored such that lower scores reflect poorer health status (i.e., more fatigue); 6MWT, a measure of distance walked, reflects scores in meters/feet (e.g., the distance a healthy individual can walk on a hard, flat surface in six minutes is approximately 400–700 m (or approximately 1300–2300 feet) [30] with lower scores indicating worse level of physical activity; EQ- 5D VAS (range = 0–100) is scored such that lower scores reflect poorer health status; the 5XSST measures the time it takes an individual to stand up and sit down five times in a row with higher times indicative of poorer health status; the SWAY quantifies postural sway from 0 to 100 with lower scores indicative of greater instability/poorer health status; Activity Duration and Activity Intensity, as assessed by accelerometry data collected from the AVIVO™ Mobile Patient Management System, reflects patient activity/mobility with lower scores indicative of poorer health; each of the CGI-S, PGI-S, CaGI-S, 6MWT PRO fatigue, and 6MWT PRO dyspnea measure concept severity with higher scores indicative of poorer health status;

*Pearson correlations

**Spearman correlations

In general, BTHS-SA domain scores were moderately to strongly related to other patient reported measures of fatigue (e.g., r = 0.59, 0.76, 0.68, and 0.61 between the PROMIS Fatigue SF and the 2 FS, 3 FS, 4 FS, and 5MS, respectively) and overall symptom severity (e.g., r = 0.60, 0.62, 0.56, 0.53 between the PGI-S and the 2 FS, 3 FS, 4 FS, and 5MS, respectively). Moreover, the magnitude of these relationships, as expected, was stronger than what was observed with the BTHS-SA scales and clinician reports of symptom severity (e.g., r = 0.40, 0.40, 0.54, and 0.36 with the CGI-S and the 2 FS, 3 FS, 4 FS, and 5MS, respectively) and functional assessments (e.g., r = − 0.30, − 0.47, − 0.52, and − 0.51 with the 6MWT and the 2 FS, 3 FS, 4 FS, and 5MS, respectively). It is also important to note the marginally weak relationship between the BTHS-SA and the EQ5D VAS (r = − 0.28, − 0.34, − 0.32, and − 0.23 and the 2 FS, 3 FS, 4 FS, and 5MS, respectively). This is an expected result given the concepts assessed by the BTHS-SA scales (i.e., fatigue and myopathy symptoms) and EQ- 5D VAS (overall health status) are more distally related. Nevertheless, the direction of the relationship—lower symptom severity associated with greater overall health ratings—was as expected.

The correlational results in Table 4 are presented to evaluate the relationship of observed change in BTHS-SA scales scores with change from reference measures. Change scores from a target measure that fluctuate in concert with changes observed in other measures as expected suggest that assessment is sensitive or sensitive to change. Similar to the cross-sectionally determined correlations, a pattern of anticipated relationships was observed.

**Table 4:** Correlations of BTHS-SA change scores with reference measure change scores

Measure	2 FS	3 FS	4 FS	5MS
	r	r	r	r
PROMIS fatigue SF*	0.74	0.66	0.59	0.59
6MWT distance*	− 0.53	− 0.55	− 0.57	− 0.57
EQ- 5D VAS*	− 0.50	− 0.46	− 0.50	− 0.48
5XSST*	0.27	0.26	0.29	0.28
Leg strength*	− 0.57	− 0.44	− 0.43	− 0.42
SWAY*	− 0.55	− 0.59	− 0.54	− 0.47
Activity duration*	− 0.42	− 0.34	− 0.19	− 0.18
Activity intensity*	− 0.42	− 0.34	− 0.18	− 0.17
CGI-S (overall item)**	0.36	0.32	0.36	0.28
CGI-C (overall item)**	0.56	0.54	0.49	0.46
PGI-S (overall item)**	0.73	0.77	0.67	0.61
PGI-C (overall item)**	0.81	0.79	0.82	0.80
CaGI-S (overall item)**	0.65	0.64	0.58	0.58
CaGI-C (overall item)**	0.59	0.64	0.66	0.66
6MWT PRO fatigue (post)**	0.42	0.39	0.50	0.48
6MWT PRO dyspnea (post)**	0.48	0.44	0.45	0.40

To facilitate interpretation, each of the 2 FS (range = 2–10), 3 FS (range = 3–15), 4 FS (range = 4–20), and 5MS (range = 5–25) are scored such that higher scores reflect poorer health status; PROMIS Fatigue SF (range = 0–52) is scored such that lower scores reflect poorer health status (i.e., more fatigue); 6MWT, a measure of distance walked, reflects scores in meters/feet (e.g., the distance a healthy individual can walk on a hard, flat surface in six minutes is approximately 400–700 m (or approximately 1300–2300 feet) [30] with lower scores indicating worse level of physical activity; EQ- 5D VAS (range = 0–100) is scored such that lower scores reflect poorer health status; the 5XSST measures the time it takes an individual to stand up and sit down five times in a row with higher times indicative of poorer health status; leg strength, as measured by HHD, reflects pounds or kilograms of force exerted by an individual upon muscle contraction with lower scores indicative of less strength; the SWAY quantifies postural sway from 0 to 100 with lower scores indicative of greater instability/poorer health status; Activity Duration and Activity Intensity, as assessed by accelerometry data collected from the AVIVO™ Mobile Patient Management System, reflects patient activity/mobility with lower scores indicative of poorer health; each of the CGI-S, PGI-S, CaGI-S, 6MWT PRO fatigue, and 6MWT PRO dyspnea measure concept severity with higher scores indicative of poorer health status; and each of the CGI-C, PGI-C, and CaGI-C assess perceived change from study baseline with lower scores indicative no change in health status

*Pearson correlation

**Spearman correlation

Using the same interpretative guidelines as above, changes in BTHS-SA domain scores were moderately to strongly related to change observed on patient reports of symptom severity either via the PGI-S (r = 0.73, 0.77, 0.67, and 0.61 for the 2 FS, 3 FS, 4 FS, and 5MS, respectively) or as reported directly by the patient via the PGI-C at the end of treatment (r = 0.81, 0.79, 0.82, and 0.80 for the 2 FS, 3 FS, 4 FS, and 5MS, respectively). Moreover, the magnitude of these relationships was larger than those observed between BTHS-SA change scores and changes observed by either the clinicians (e.g., via the CGI-S and CGI-C) or functional assessments (e.g., 6MWT). Interestingly, while not as strong in magnitude as between the change in BTHS-SA scale scores and other patient reports, the relationship between change in BTHS-SA scale scores and caregiver reports of change (e.g., r = 0.65, 0.64, 0.58, and 0.58 with the CaGI-S and r = 0.59, 0.64, 0.66, and 0.66 with the CaGI-C for the 2 FS, 3 FS, 4 FS, and 5MS, respectively) were stronger than with either the clinician and functional assessments.

## Discussion

Establishing the reliability and validity of new PRO measures is a critical step in supporting their use in clinical trials. The results presented here suggest that the BTHS-SA can produce reliable scores and, moreover, that those scores provide a valid reflection of symptom experience when administered to patients with BTHS participating in a clinical study.

The initial psychometric results presented here are important for several reasons. First, there are no patient-centered measures that have been specifically developed to assess BTHS symptoms. The BTHS-SA was developed to address this unmet need and results of this evaluation, along with its content validity evidence [13], not only give BTHS researchers a way to directly assess the BTHS symptom experience but also justifies its use to evaluate treatment benefit among individuals with this ultra-rare disease.

Secondly, the results presented here support the reliability of BTHS-SA scores and the validity of the conclusions drawn from them in a previously reported clinical trial [17]. In the TAZPOWER study, change from baseline on the BTHS-SA Total Fatigue Score (the 4 FS as referenced in the current analysis) was a primary endpoint and although statistically significant improvement was not observed in the randomized, controlled segment of the trial, the Total Fatigue Score/4 FS was statistically significantly reduced from baseline among patients in the open label extension (OLE).

BTHS is an ultra-rare disease and, accordingly, the sample size used in the analysis presented here was small. As a result, it is difficult to draw definite conclusions from these results; small samples yield statistics (such as group means and correlations) that are subject to considerable sampling error. Due to this, and given that ongoing and interrelated psychometric inquiries are essential to understanding the reliability and validity of any PRO measure (particularly for assessments early in development), additional research with this underserved population is needed to continue to establish the measurement characteristics of the BTHS-SA as well as how to interpret the meaning of observed scores. Dependent on factors like sample size, future research could consider data analytic choices relevant to modern measurement theory (e.g., use of Rasch models) or classical test theory such as those used here. Additionally, and given the ultra-rarity of the disease, researchers can consider designing studies allowing for a normal, age-matched population to facilitate certain aspect of psychometric evaluation [31].

It is difficult to recommend one of the fatigue scale configurations for use in future research, due to the small sample size and overlapping findings across analyses in this study. The difference between the 2 FS, 3 FS, and 4 FS involves the inclusion of items assessing tiredness and muscle weakness “at rest,” which could be an indicator of severe problems with fatigue. Including these items could be useful if a study will enroll participants with severe disease-related symptoms and limitations to increase the sensitivity of the fatigue score. Adding complementary measures of other relevant concepts (e.g., disease-related impacts on functioning, such as difficulty holding conversations with others) and clinical measures (e.g., cardiac medication use) will also be useful in future research.

## Conclusion

The results of this psychometric evaluation of the BTHS-SA as used in the TAZPOWER study demonstrate that the assessment is capable of producing reliable scores upon which valid inferences may be drawn when administered among patients with BTHS. Researchers can use the BTHS-SA to evaluate novel treatment benefits. Eventually, it may be used to inform healthcare decisions and improve patients’ lives in real-world clinical practice. Because of the small sample sizes associated with the rarity of this condition in this study, and the need for replication of psychometric results, the conclusions drawn here should be confirmed in future studies with this underserved patient population.

## Data Availability

The TAZPOWER data sets generated and analyzed during the study are not publicly available but are available from the Hilary J. Vernon (hvernon1@jhmi.edu) upon request.

## Abbreviations

2 FS: Two-item fatigue score
3RS: Three-item fatigue score
4 FS: Four-item fatigue score
5MS: Five-item myopathy score
5XSST: Five times sit-to-stand test
6MWT: Six-minute walk test
BTHS: Barth syndrome
BTHS-SA: Barth syndrome symptom assessment
CaGI-C: Caregiver global impression of change
CaGI-S: Caregiver global impression of severity
CGI-C: Clinician global impression of change
CGI-S: Clinician global impression of severity
HHD: Handheld dynamometer
ICC: Intraclass correlation coefficients
OLE: Open label extension
PGI-C: Patient global impression of change
PGI-S: Patient global impression of severity
PRO: Patient-reported outcome
PROMIS: Patient reported outcome measurement information system
SD: Standard deviation
SWAY: SWAY application balance assessment
TAZ: TAFAZZIN
